# Electrostatic potential difference between tumor and paratumor regulates cancer stem cell behavior and prognose tumor spread

**DOI:** 10.1002/btm2.10399

**Published:** 2022-09-23

**Authors:** Haoran Zhao, Weijie Zhang, Xiaowei Tang, Edgar A. Galan, Yinheng Zhu, Gan Sang, Davit Khutsishvili, Honghui Zheng, Shaohua Ma

**Affiliations:** ^1^ Tsinghua Shenzhen International Graduate School (SIGS) Tsinghua University Shenzhen China; ^2^ Tsinghua‐Berkeley Shenzhen Institute (TBSI) Shenzhen China; ^3^ Department of Oncology The First Affiliated Hospital, Zhengzhou University Zhengzhou China; ^4^ Shenzhen Bay Laboratory Shenzhen China

**Keywords:** electrostatic potential, cancer prognosis, cancer stem cells, paratumor, tumor, tumor spread

## Abstract

Tumor spread is responsible for most deaths related to cancer. Increasing the accuracy of cancer prognosis is critical to reducing the high mortality rates in cancer patients. Here, we report that the electrostatic potential difference (EPD) between tumor and its paratumor tissue is a prognostic marker for tumor spread. This finding is concluded from the patient‐specific EPD values and clinical observation. The electrostatic potential values were measured on tissue cryosections from 51 patients using Kelvin probe force microscopy (KPFM). A total of ~44% (15/34) patients of V_tumor–paratumor_ > 0 were featured with tumor spread, whereas only ~18% (2/11) patients of V_tumor–paratumor_ < 0 had tumor spread. Next, we found the increased enrichment of cancer stem cells in paratumors with lower electrostatic potentials using immunofluorescence imaging, which suggested the attribution of tumor spread to the galvanotaxis of cancer stem cells (CSCs) toward lower potential. The findings were finally validated in breast and lung spheroid models composed of differentiated cancer cells and cancer stem cells at the ratio of 1:1 and embedded in Matrigel dopped with negative‐, neutral‐ and positive‐charged polymers and CSCs prefer to spread out of spheroids to lower electrostatic potential sites. This work may inspire the development of diagnostic and prognostic strategies targeting at tissue EPDs and CSCs for tumor therapy.

## 1 INTRODUCTION

Metastasis or tumor spread accounts for about 90% of deaths related to cancer.^1^ Currently, there are mainly two competing hypotheses to explain tumor progression.^2^ The stochastic model considers that all tumor cells can mutate and become tumorigenic. The hierarchical stem cell model suggests that only a distinct subpopulation of cells, termed cancer stem cells (CSCs) and originated at the time of tumor initiation are capable of metastasis. Several biomarkers that characterize CSCs have been identified and linked to prognostic and therapeutic outcomes.^3^ However, the characterization of CSCs based on their physical properties have received less attention, which limits the diversity and accuracy of CSC identification in practice.

Electric fields regulate cell proliferation,^4^ differentiation,^5^ migration,^6^ and various other important biological processes,^7^, ^8^ though the underlying mechanisms remain unclear.^9^, ^10^ It has been suggested that exogenous electric fields can affect the cell's transmembrane potential,^11^ by altering the activity of membrane ion channels.^12^ Interestingly, highly proliferative cells have a significantly depolarized membrane potential compared to nonproliferating cells.^8^ Additionally, membrane depolarization inhibits stem cell differentiation^4^ and plays a crucial role in cytoskeletal rearrangement,^13^ which is required for both mitosis and cell migration. Therefore, it is plausible that the inherently different bioelectric properties of the tumor microenvironment can promote the maintenance and spread of CSCs. For example, galvanotaxis of breast cancer cells (4T1) was demonstrated on the application of physiological levels of electric fields in vitro.^14^

Electrical stimulation has been extensively investigated for its potential to modulate immune responses^15^ and destroy cancer cells^16^, ^17^ (including CSCs^18^). However, the use of electrostatic potentials for CSC identification, quantification, and migratory studies remains underexplored. Devising the bioelectric nature of CSCs and understanding their behavior under electrostatic gradients could provide physical markers^19^, ^20^, ^21^, ^22^, ^23^ to enable a more robust distinction and preferential administration between CSCs and differentiated tumor cells. The integration of such physical markers into current practices for tumor evaluation may strengthen cancer prognosis and treatment.

**FIGURE 1:**
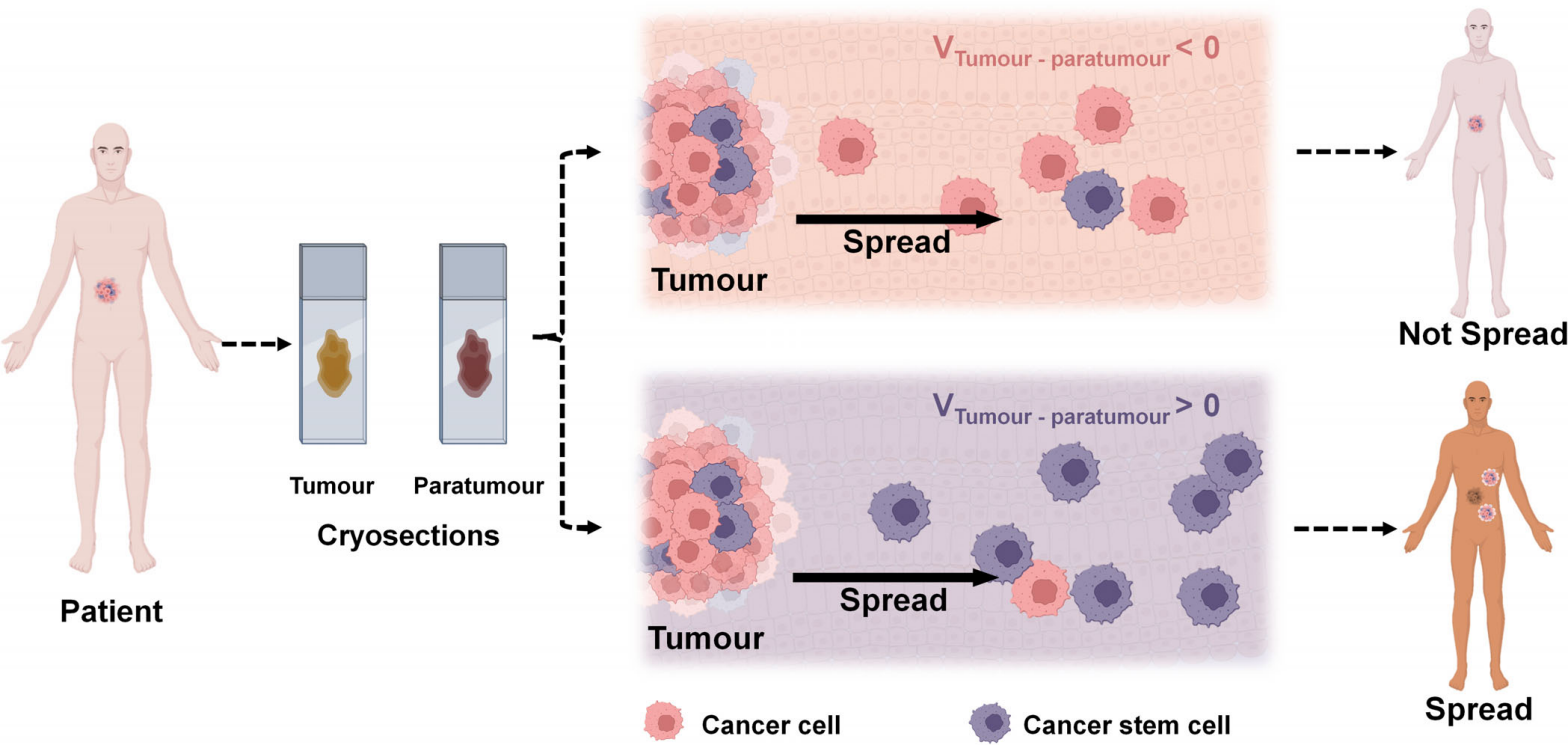
Schematic diagram depicting the finding that tumor spread is more likely to occur when the electrostatic potential between tumor and paratumor tissues (V_Tumor–Paratumor_) is positive

Here, we investigated the correlation between tumor spread and tumor‐to‐paratumor EPD by using both patient tissues and in vitro models. We found that tumor cryosections with a positive potential difference correlated with higher rates of tumor spread in patients (Figure 1) and higher expression of CD44—a known CSC marker.^24^ We also demonstrated the directed migration of CSCs toward regions of low electrostatic potential in vitro. We, therefore, propose that the tumor‐to‐paratumor electrostatic potential difference (EPD), which can be measured on cryosections of tumor biopsy, is prognostic of tumor spread.

## 2 RESULTS

### 2.1 Tumor–paratumor EPD shows a high correlation with chances of tumor spread

**FIGURE 2:**
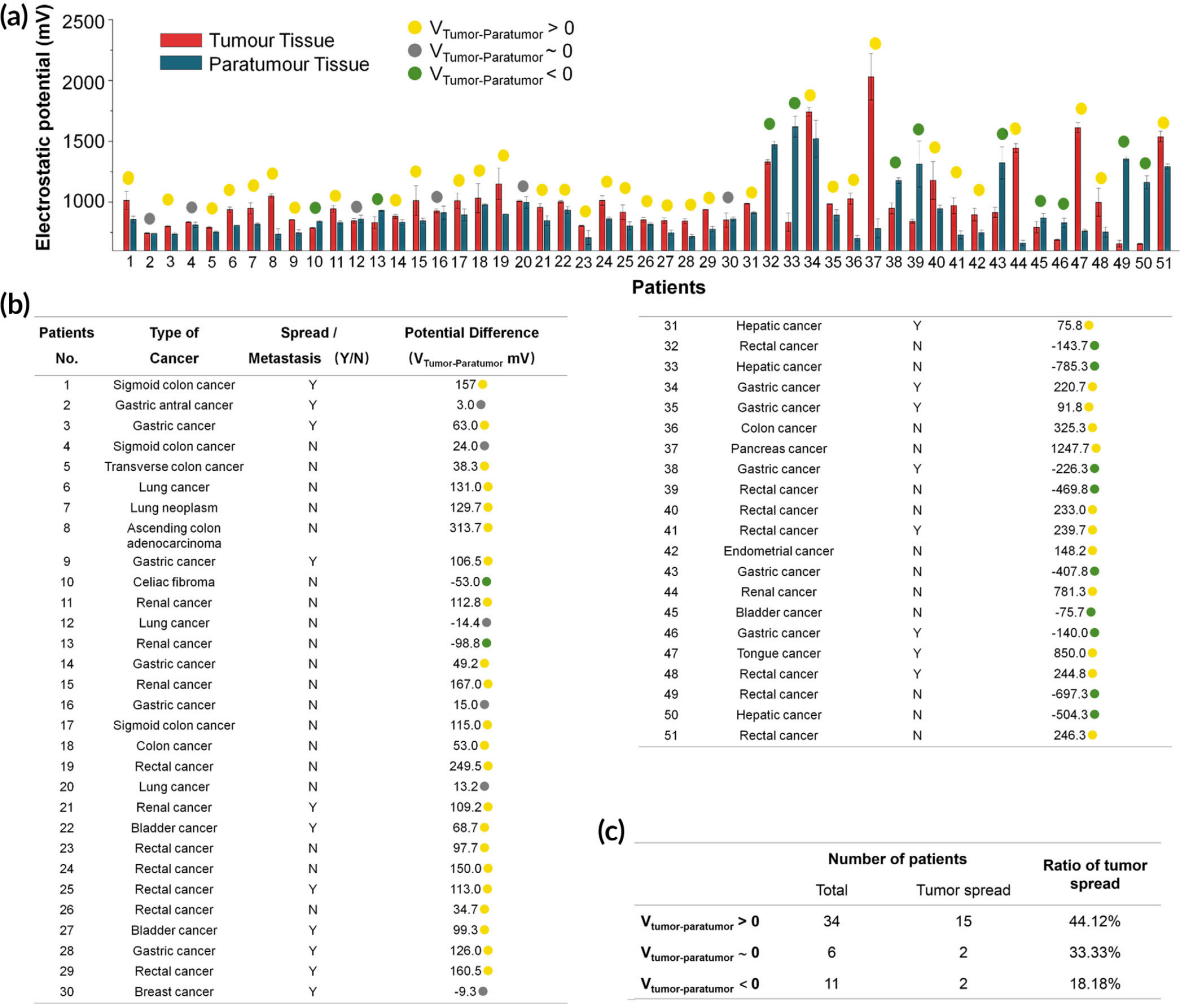
Patient sample data and electrostatic potential differences. (a, b) Electrostatic potential differences between tumor and paratumor cryosections (V_Tumor–Paratumor_) were measured using a Kelvin probe force microscope (KFPM). Samples with a positive, negative, or negligible potential difference are denoted by yellow, green, or gray dots, respectively. Data are presented as the mean value ± standard deviation of five measurements taken at different locations. (c) Samples are grouped according to the electrostatic potential difference.

We hypothesized that tumor and paratumor tissues could have different electrostatic potentials in most types of tumors and that these potentials could be measured in cryosections and may correlate with tumor spread in patients. We, therefore, collected tumor and paratumor tissue samples from 51 patients, comprising 16 different types of cancers classified as Grades 1–3, some of which had and had not spread (Table S1). We first prepared tumor and paratumor tissue cryosections and measured the electrostatic potentials using a Kelvin probe force microscope (KPFM)^25^ (Figure S1). We then compared the electrostatic potentials between the tumor and paratumor regions and grouped the samples according to potential differences and whether tumors had spread. These data suggest that tumors were more likely to spread when the electrostatic potential was higher in the tumor than in the paratumor tissues, because 15 out of 34 (44%) patients with V_Tumor–Paratumor_ > 0 were featured with tumor spread, including metastasis. However, the chance of tumor spread was much reduced when the electrostatic potential was lower in the tumor because only 2 out of 11 (18%) patients of V_Tumor–Paratumor_ < 0 had tumor spread. The remanent six patients of the study cohort were V_Tumor–Paratumor_ ~ 0, among which, two tumors spread. The percentage of spread was the intermediate of the former two groups (Figure 2).

**FIGURE 3:**
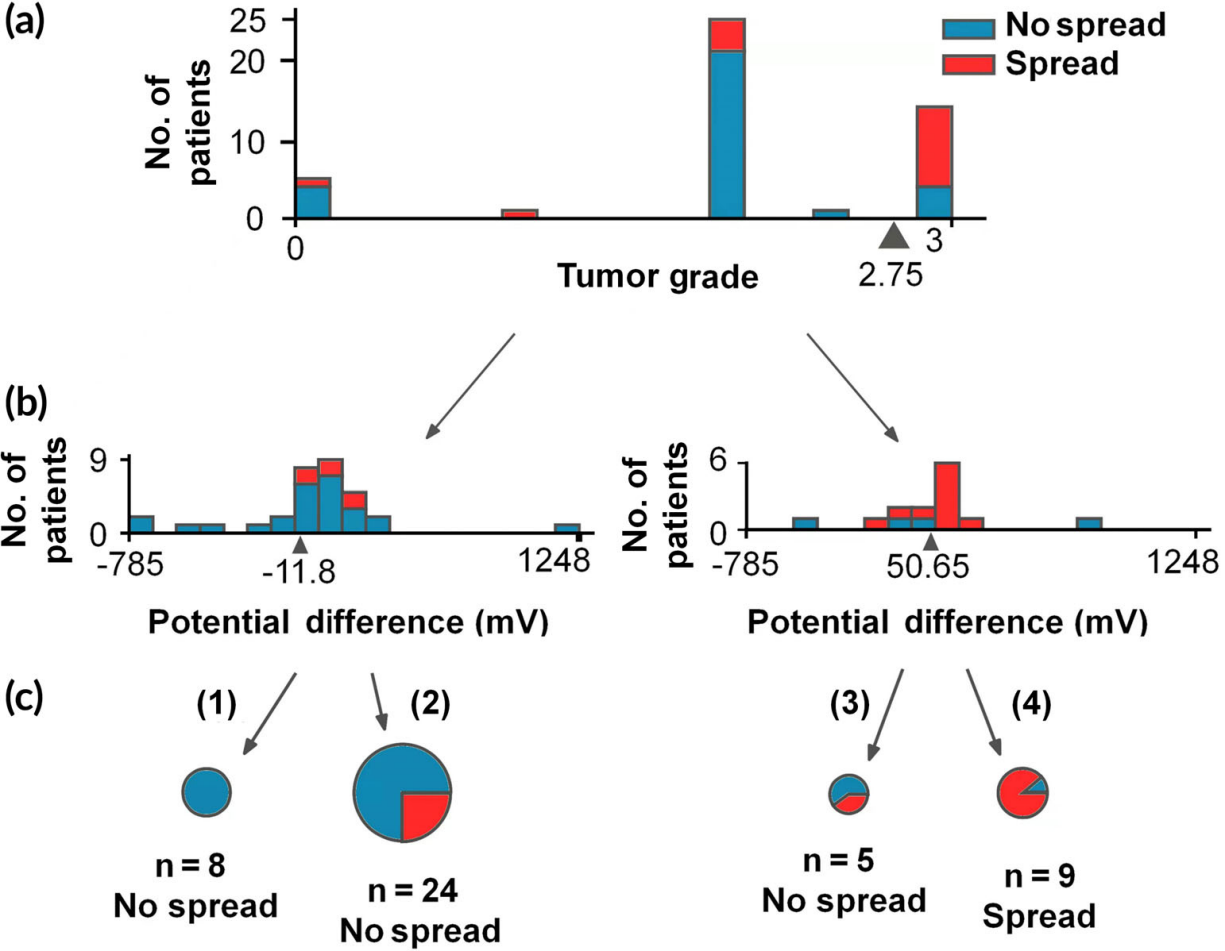
Patient data grouped based on tumor grading and EPD (V_tumor–paratumor_). By setting thresholds on grade 2.75 (a) and on potential differences −11.8 and 50.56 mV (b), the study cohorts were classified into four groups (1–4, C). Group (1, EPD < −11.8 mV) and Group (4, EPD > 50.65 mV) displayed high correlation between EPD and tumor spread

We next included the tumor grades data to further categorize the samples into four groups: (1) V_Tumor–Paratumor_ < −11.8 mV and grade <2.75, (2) V_Tumor–Paratumor_ > −11.8 mV and grade <2.75, (3) V_Tumor–Paratumor_ < 50.65 mV and grade >2.75, and (4) V_Tumor–Paratumor_ > 50.65 mV and grade >2.75 (Figure 3). The C4.5 algorithm was used to perform fitting (parameter estimation). It is known from the previous step that the splitting threshold in a point inside the highlighted interval could reach optimal Gini impurity. The final splitting threshold would be the arithmetic average of boundary values. In this study, the splitting threshold became (2.5 + 3) × 0.5 = 2.75. By using the same method, the splitting threshold for EPD was obtained at −11.8 and 50.65. We found that these values provided a high confidence correlation for tumor prognosis; for Group 1, all 8 (100%) tumors were reported nonspread, whereas, for Group 4, eight out of nine (89%) tumors had spread (Table S2). In Figure 3, the percentages of tumor spread in Groups 2 and 3 were 25% (6/24) and 40% (2/5), respectively. Groups 2 and 3 showed a weak correlation between tumor spread and V_Tumor–Paratumor_.

### 2.2 CSCs are enriched in paratumors of lower electrostatic potential

**FIGURE 4:**
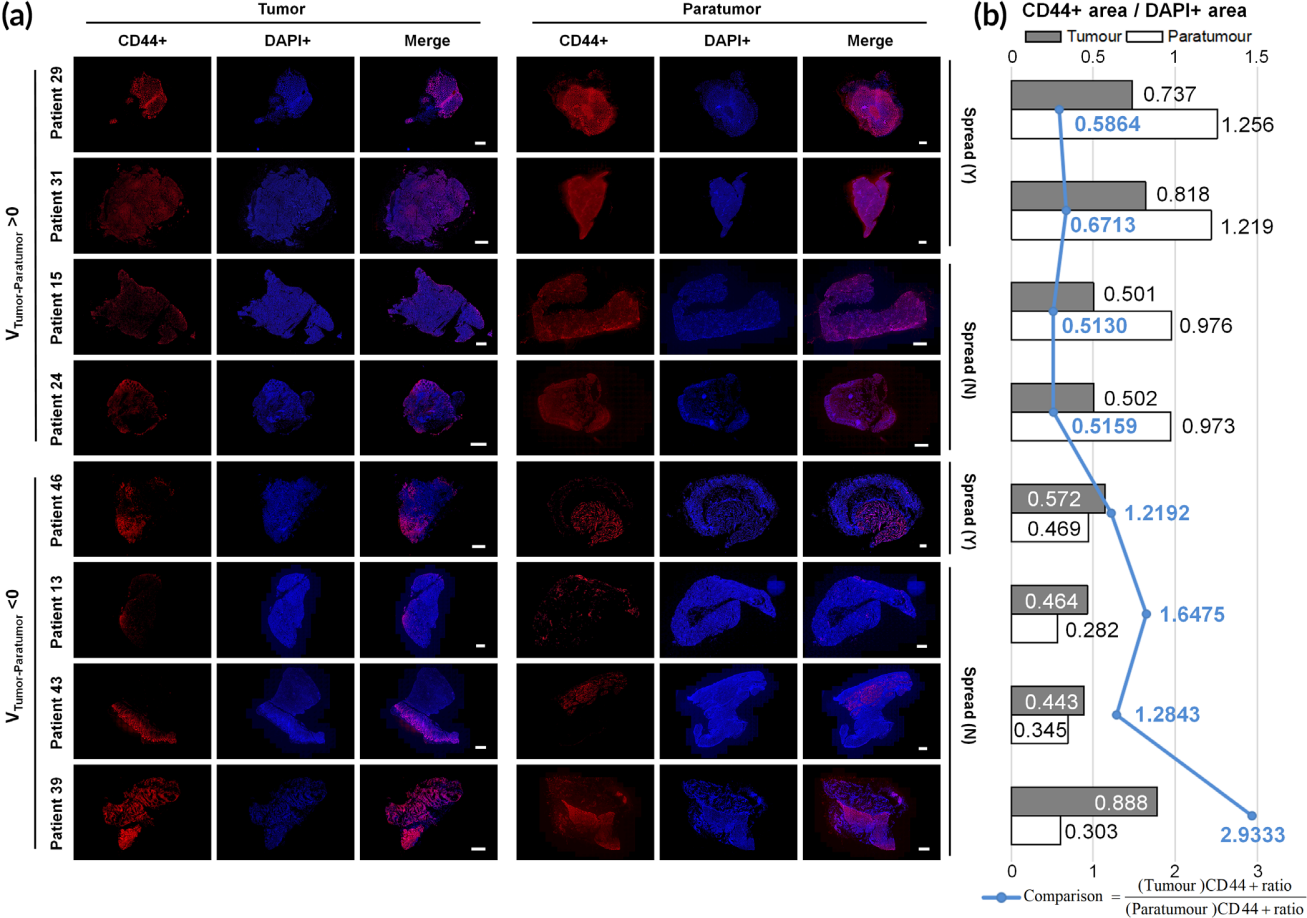
Fluorescence microscopy of tumor and paratumor tissue cryosections stained for the CSC marker CD44. (a) Immunofluorescence imaging reveals the CSC distribution in tumor and paratumor tissues. (b) Quantification of the stained CD44+ area to the DAPI+ area in (a). The blue line indicates the ratio of CD44+ area between tumor and paratumor tissues. Ratios were lower when V_Tumor–Paratumor_ > 0 mV and higher when V_Tumor–Paratumor_ < 0 mV, independent of whether tumors had spread. Scale bar: 1 mm. *p < 0.05

Next, eight patient samples were chosen at random to investigate the spatial distribution of CSCs in tumor and paratumor tissues. Cryosections were prepared and stained for the CSC marker CD44 and examined under a fluorescent microscope (Figure 4). Other reported CSC markers were investigated, including GD2,^26^ CD133, CD24, and ANGPTL4.^27^ CD44 was chosen because of its prevalence in pan‐cancer CSCs. Dual‐ or triple staining was not adopted to avoid the high occurrence rate of false negatives. It was found that CSCs were significantly more abundant in the paratumor tissues of all the four samples where V_Tumor‐Paratumor_ > 0 mV and were less abundant in all the four paratumor samples where V_Tumor–Paratumor_ < 0 mV. In either group, the spread‐tumor had higher enrichment of CSCs in its paired paratumor compared with the nonspread tumor, suggesting a positive correlation between CSC enrichment and tumor spread. The increased CSC enrichment may be attributed to directed CSC migration toward lower potential regions or elevated proliferation under lower potential stimulation. It is worth further exploration to elaborate on the mechanism.

### 2.3 Directional migration of CSCs in charged Matrigel

**FIGURE 5:**
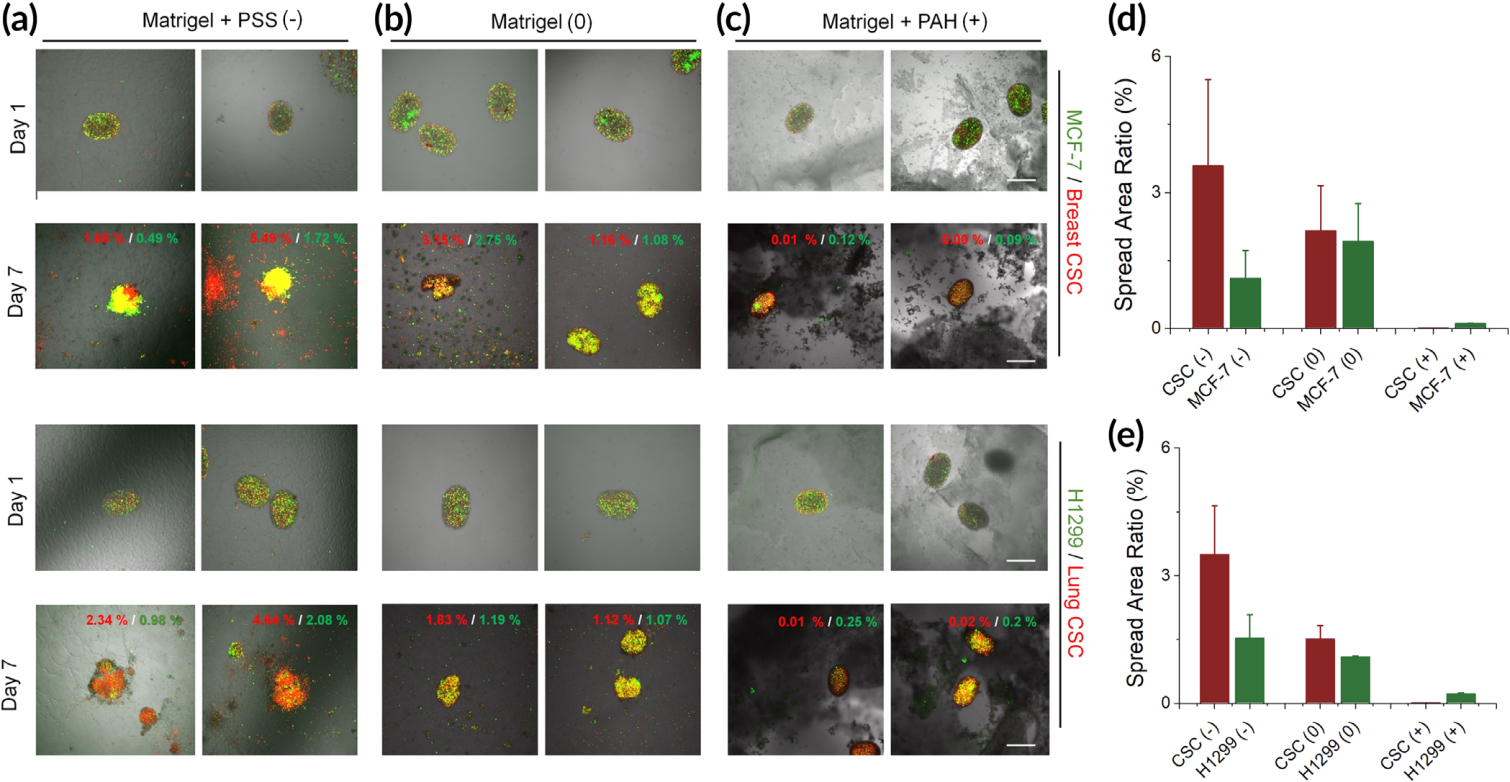
Directional migration of breast (MCF‐7) and lung (H1299) cancer cells and CSCs in charged Matrigel. (a–c) Tumor spheroids were seeded in six‐well plates coated with (b) Matrigel or a mix of Matrigel and (a) negatively (polystyrene sulfonic acid [PSS]) or (c) positively charged polymers (polyallylamine hydrochloride [PAH]) and imaged on Days 1 and 7 under a fluorescence microscope. Red digits in the images: the area ratios of red fluorescence (CSCs) out of the entire imaging windows; green digits: the area ratios of green fluorescence (differentiated cancer cells) out of the entire imaging windows. (d, e) Summarized the area ratios (fraction) in (a–c) at Day 7. Scale bar: 500 μm. *n* = 4

Next, we established tumor spheroid models to investigate their responses toward different electrostatic potentials, that is, galvanotaxis. Charged polymers, polystyrene sulfonic acid (PSS), and polyallylamine hydrochloride (PAH) were employed to dop Matrigel at 1 wt% of the parental scaffold, rendering it negative and positive, respectively. The spheroids were composed of CD44‐stained CSCs and differentiated cancer cells at a 1:1 ratio and embedded in Matrigel mixed with or without electrostatically charged polymers to simulate the lower, higher, and neutral electrostatic potentials in the peripheral environment (Figure 5). Two tumor models, breast and lung, were established (Figure 5, Figures S2 and S3). Both breast and lung CSCs showed strong directional migration toward the negatively charged substrate but not toward the neutral or positively charged ones. At Day 7, significantly higher rates of CSCs than differentiated cancer cells (more than twofold higher of CSCs over differentiated breast/lung cancer cells) migrated toward the Matrigel substrate doped with PSS, but the rates reached nearly identical in neutral Matrigel and approached 0 in Matrigel doped with PAH. These data verified the hypothesis that CSCs tend to migrate toward regions of negative electrostatic potential.

## 3 DISCUSSION

Precise staging and prediction of cancer progression aim to reduce the burden of cancer‐related mortality. The different bioelectric properties of differentiated cancer cells and CSCs can be used to develop new prognostic tools for cancer spread. In this study, we investigated the correlation between Tumor–Paratumor EPD (V_Tumor–Paratumor_) and tumor spread. We hypothesized that V_Tumor–Paratumor_ drives tumor spread primarily by facilitating the migration of CSCs. Hence, we studied the distribution of CD44+ CSCs in patient tissue with a positive and negative V_Tumor–Paratumor_ and the directional migration of CSCs and cancer cell lines (MCF‐7 and H1299 for breast and lung modeling, respectively) from simulated spheroid models to the embedding charged Matrigel substrate.

Our results show that tumor spread has a strong correlation with the EPD between tumor and paratumor, and patients with positive V_Tumor–Paratumor_ have higher rates to develop tumor spread. This is consistent with a recent study that showed that high tumor potential value was correlated with the advanced stage of epithelial ovarian cancer.^28^ Additionally, CD44+ CSCs showed directional migration toward the negatively charged substrate. We also observed that a higher abundance of CD44+ CSCs was observed in patient paratumor tissue when V_Tumor–Paratumor_ was positive. CSCs present in metastatic tumors have a relatively depolarized (less negative) membrane potential as compared to differentiated cancer cells.^13^ Together, these results suggest that tumor spread is facilitated toward areas of lower electrostatic potential. Thus, the electric potential of tumor and paratumor tissues regulates cancer stem cell behavior and shows a high correlation with tumor spread. It could potentially be developed as a prognostic biomarker to approximate the extent of tumor spread and assist medical personnel in choosing appropriate therapies. A biopsy‐like tool functionalized with sensing modules for electrostatic potential (or potential difference along the biopsy axis) may allow direct measurement of V_Tumor–Paratumor_. It, thus, enables facile prediction on tumor spread and decision‐making of therapies. A needle tool with negative charge coating may serve to enrich CSCs that benefit tumor diagnosis, because CSCs tend to approach negative potentials.

This work provides an initial framework of electrostatic potential‐driven tumor spread upon which future studies can be built. Probing tools might be developed to reach in situ investigation of tumor and paratumor electrostatic potentials. However, this study has limitations. First, more tissue slices should be examined to provide the statistical power to consolidate conclusions on CSC distribution and tumor spread. A prospective study from a larger cohort size is required to validate that a positive V_Tumor–Paratumor_ is a prognostic marker of tumor progression. Second, different cells show different electrotaxis^29^, ^30^, ^31^, ^32^ and the bioelectric properties of CSCs and differentiated cancer cells must be elucidated to understand and apprehend the directional movement of CSCs. Till then, the prognostic promise of EPD might benefit personalized cancer therapy. Before its implementation, large cohort clinical studies must be performed to validate its prognostic ability, including both retrospective and prospective examinations.

## 4 METHODS

### 4.1 Tumor tissue grading and sample collection

Tumor biopsies were visually examined under a microscope using hematoxylin and eosin (H&E) staining by doctors at the First Affiliated Hospital, Zhengzhou University. Tumors were classified from Grades 1–3 according to the appearance of tumor cells as (1) well‐differentiated when tumor cells were similar to those of the surrounding normal tissue; (3) tumor cells were poorly differentiated; or (2) a state in between Grades 1 and 2. Tumor and paratumor biopsies (3–5 mm away from the primary tumor) were obtained from the Department of Oncology, The First Affiliated Hospital, Zhengzhou University, under the guidelines of the hospital ethics committee and with informed consent from patients. Samples were frozen and stored and shipped at −80°C to the laboratory for further processing.

### 4.2 Preparation and immunostaining of cryosections

Tissue samples were fixed for 20 min at room temperature in 4% weight/volume (wt./vol) paraformaldehyde (PFA) in phosphate‐buffered saline (PBS) solution (Beyotime, P0099). Tissues were then washed with PBS (Thermo Fisher, 14190144), cryopreserved in 30% sucrose in PBS for 10 h at 4°C, and then transferred into the OCT embedding compound. Cryosections—16 μm for electrostatic potential measurement and 5 μm for immunostaining—were generated using a freezing microtome (Leica CM1950). Slices were immersed in double‐distilled water (ddH_2_O) for 5 min to remove soluble ions and then freeze‐dried for 5 h.

For immunostaining, tissue slices were immersed in methanol (Sigma, 34860) for 5 min and then permeabilized with 2% Triton X‐100 (Millipore Sigma, 9400) in PBS for 20 min, anti‐CD44 primary antibodies (Huabio, ET1609‐74) diluted 1:300 were added into the same solution followed by incubation overnight at 4°C. Then, primary antibodies were removed and slices were gently rinsed thrice with PBS and incubated at 37°C with Alexa Fluor Donkey anti‐Rabbit secondary antibodies (Abcam, ab175470) diluted at 1:500 for 1 h. Slices were then rinsed thrice with PBS and incubated with DAPI (Thermo Fisher, 62248) for 10 min. Images were captured using a Nikon A1R+ Laser scanning confocal microscopy.

### 4.3 Electrostatic potential measurement

The surface electrostatic potentials of cryosections were measured using a Kelvin probe force microscope (KPFM) (MDTC‐EQ‐M16‐01). A piece of gold was used as a reference. Each measurement was repeated five times at different locations.

### 4.4 Preparation of tumor spheroids and electrostatically charged Matrigel

Breast (MCF‐7) and lung (H1299) cancer cells and their corresponding cancer stem cell lines (BLUEFBIO, BFN608007236, and BFN60808718, respectively) were cultured in DMED high glucose medium (BI, 01‐056‐1A) supplemented with 20% FBS (QmSuero, mu001SR) were digested using EDTA–Trypsin (Thermo Fisher, 25200072) to obtain a single‐cell suspension, centrifuged and the supernatant discarded, then resuspended in 1 ml DMEM medium. Dilinoleyl dilinoleate (red fluorescent probe, UElandy, D4053) and DiO (green fluorescent probe, UElandy, D4007) were added to the cell suspensions (diluted 1:1000) followed by incubation at 37°C for 15 min. Cells were centrifuged and washed twice with PBS (more than 5 ml). Then, the cells were resuspended in a DMED medium and counted using a Cellometer (Thermo Fisher).

Breast CSCs mixed with breast (MCF‐7) or lung (H1299) cancer cells mixed with lung CSCs (1:1 ratio) and suspended in DMED high glucose medium (BI, 01‐056‐1A) supplemented with 20% FBS at 1 × 10^6^ cells/ml. Cells were centrifuged, resuspended in 200 μl Matrigel (Corning, 354234) and immediately injected into a T‐junction to generate monodisperse droplets.

Six‐well plates were coated with 50 μl (per well) of Matrigel (1:10 dilution) mixed with negatively or positively charged polymers, namely, 1% (w/w) polystyrene sulfonic acid (PSS; Macklin 28210‐41‐5) and polyallylamine hydrochloride (PAH; Macklin 71550‐12‐4), respectively; pure Matrigel was used as the neutral control.

## AUTHOR CONTRIBUTIONS

**Haoran Zhao:** Formal analysis (equal); investigation (equal); methodology (equal); writing – original draft (equal). **Xiaowei Tang:** Data curation (equal); methodology (equal); resources (equal); validation (equal); visualization (equal). **Weijie Zhang:** Conceptualization (equal); resources (equal); writing – original draft (equal). **Edgar A. Galan:** Writing – original draft (equal). **Yinheng Zhu:** Formal analysis (supporting); software (lead). **Sang Gan:** Formal analysis (supporting). **Davit Khutsishvili:** Writing – original draft (supporting). **Honghui Zheng:** Formal analysis (supporting).

## CONFLICT OF INTERESTS

The authors declare no competing interests.

### PEER REVIEW

The peer review history for this article is available at https://publons.com/publon/10.1002/btm2.10399.

## Supporting information


**Appendix S1** Supporting information

## Data Availability

Data available on request from the authors.
