# Supplementary material for: Electrostatic potential difference between tumor and paratumor regulates cancer stem cell behavior and prognose tumor spread
Source: Bioeng Transl Med. 2022 Sep 23;8(2):e10399. doi: 10.1002/btm2.10399 (PMC10013821; doi:10.1002/btm2.10399)
Supplement: Supplementary file 1 — Appendix S1 Supporting information [file BTM2-8-e10399-s001.docx]

# *Supplemental information*

**Electrostatic potential difference between tumor and paratumor regulates cancer stem cell behavior and prognose tumor spread**

Haoran Zhao^1,2,#^, Weijie Zhang^3,#^, Xiaowei Tang^1,2,#^, Edgar A. Galan^1,2^, Yinheng Zhu^1,2^, Gan Sang^1,2^, Davit Khutsishvili^1,2^, Honghui Zheng^1,2^, Shaohua Ma^1,2,4,5*^

^1^Tsinghua Shenzhen International Graduate School (SIGS), Tsinghua University, Shenzhen 518055, China

^2^Tsinghua-Berkeley Shenzhen Institute (TBSI), Shenzhen 518055, China

^3^Department of Oncology, The First Affiliated Hospital, Zhengzhou University, Zhengzhou 450052, China

^4^Shenzhen Bay Laboratory, Shenzhen, China

^5^Corresponding email: ma.shaohua@sz.tsinghua.edu.cn

^#^These authors contributed equally

Figure S1. Surface topography of patient sample sections obtained by AFM. P = patient.
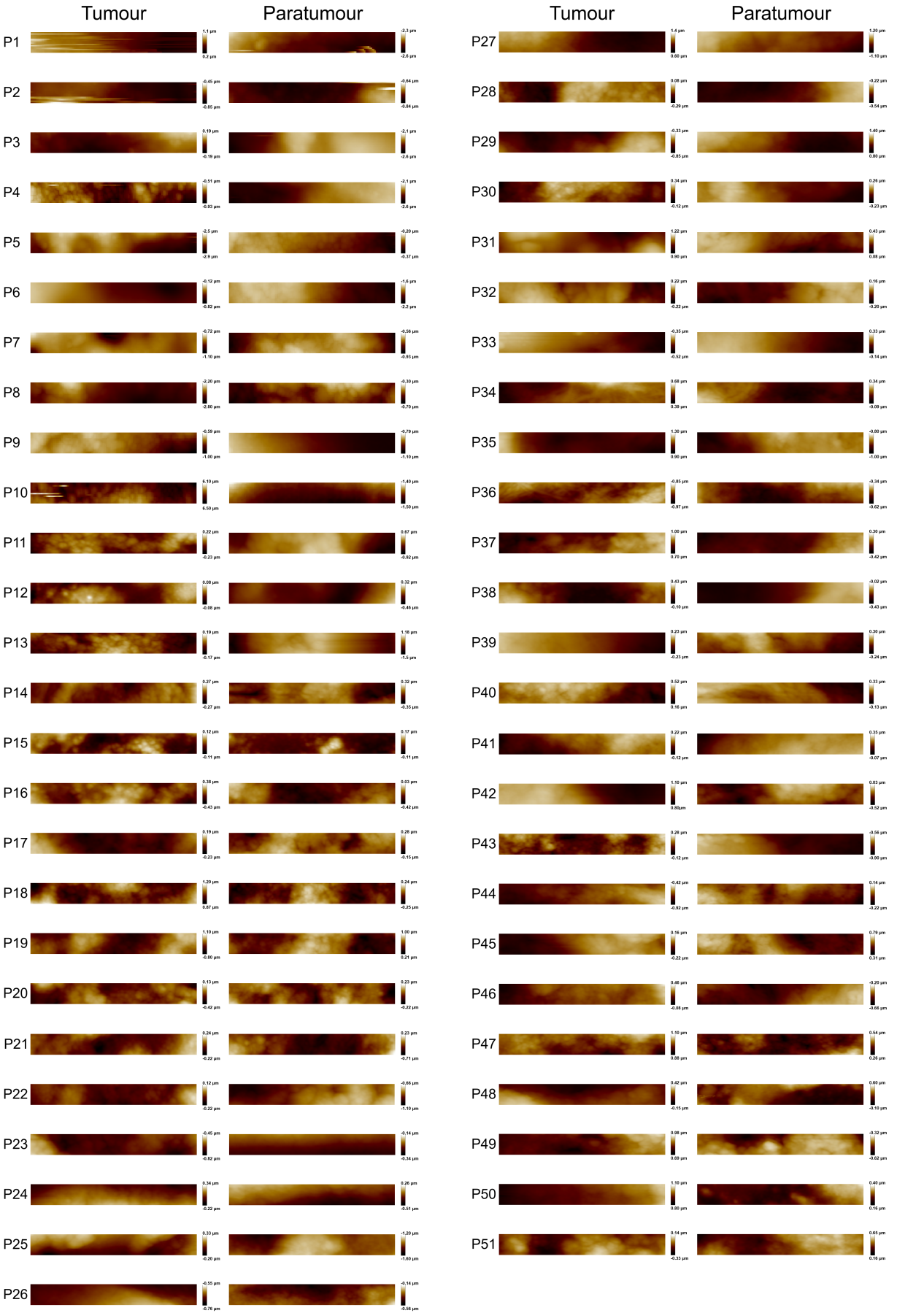


Table S1. Detailed patient information. *tumors with no grading were excluded from the statistical analyses.


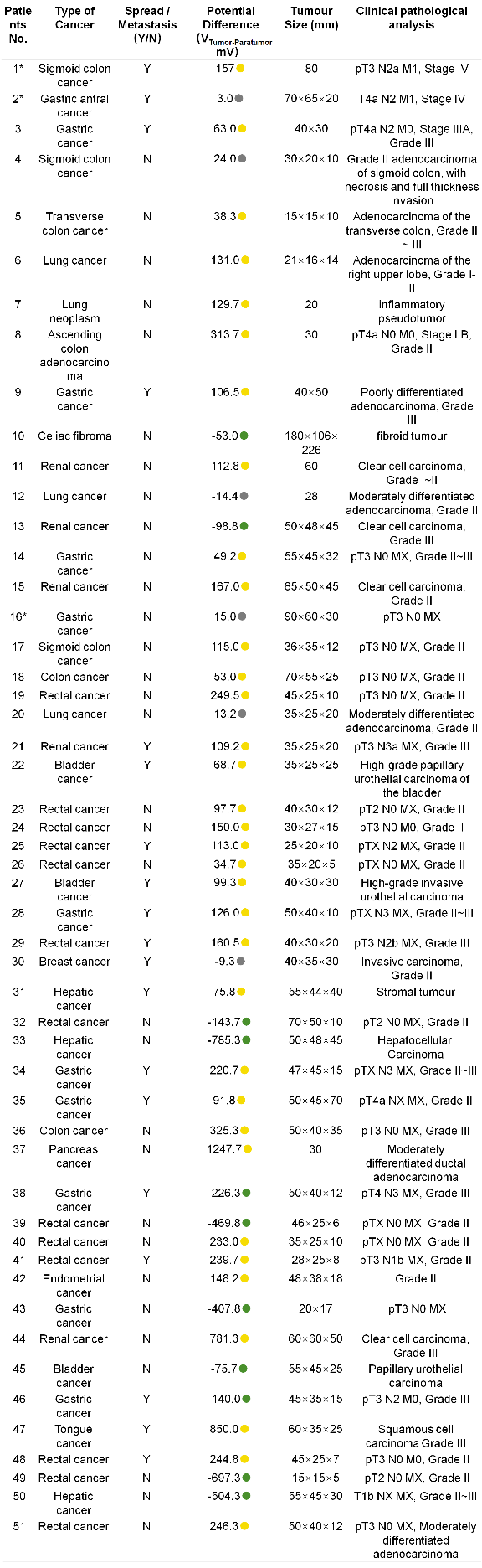


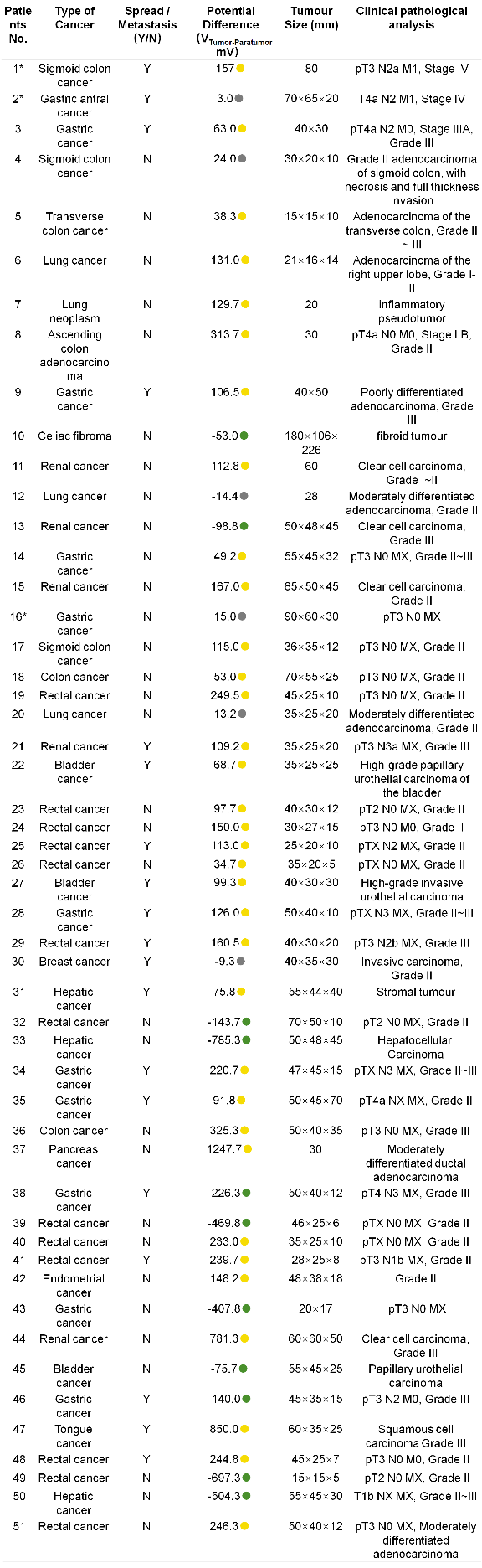


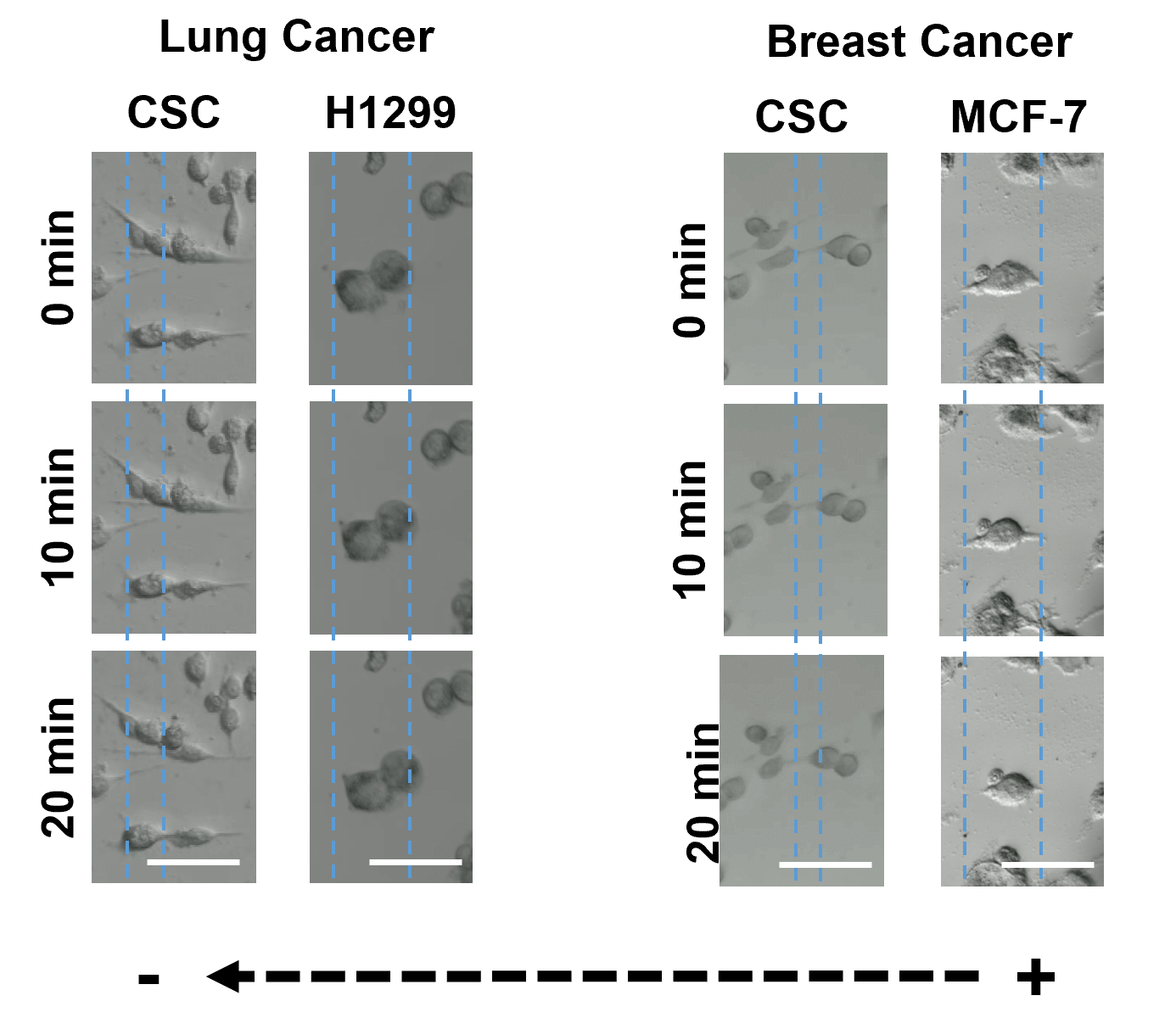


Figure S2. Directional cell spread in 2D culture under a constant electric field (100 mV/mm) for 0 min, 10 min, and 20 min. Scale bar: 50 μm.


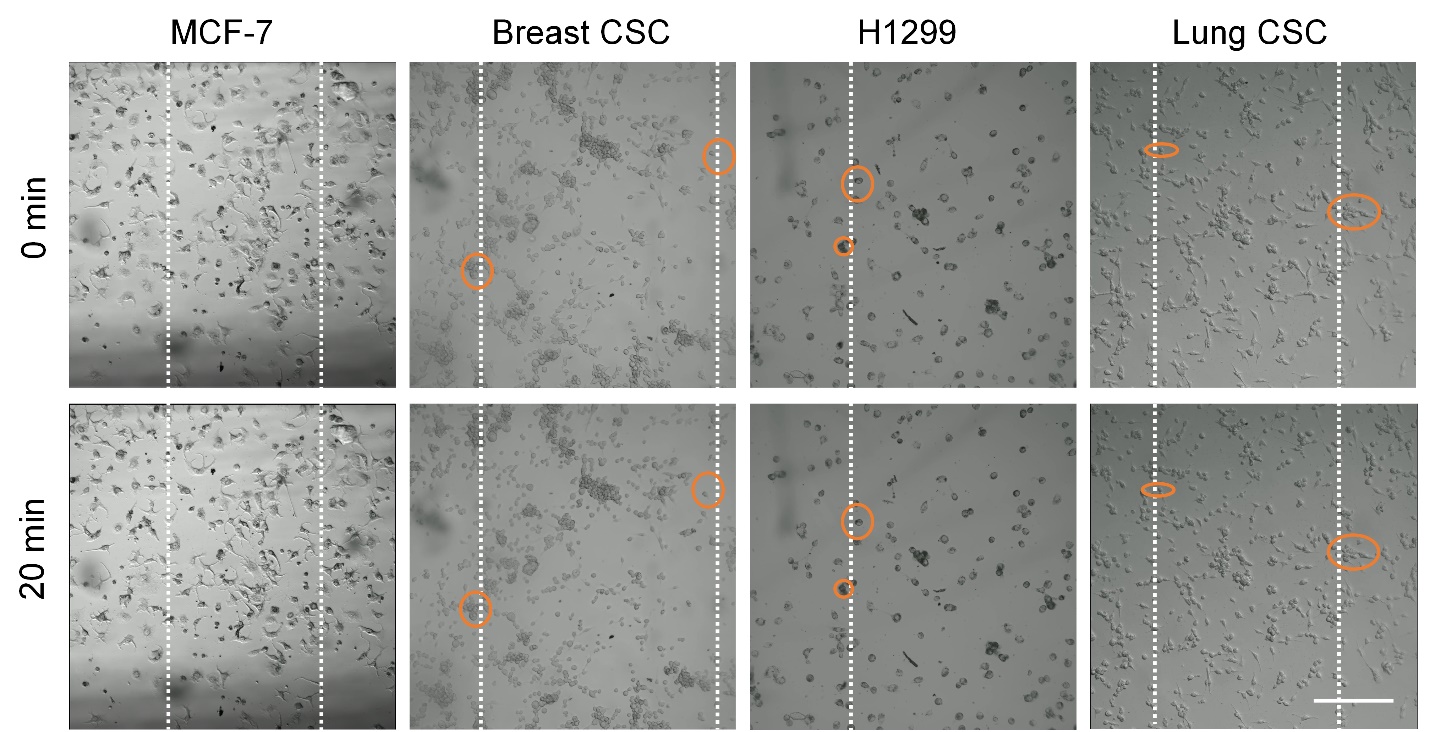


Figure S3. Directional cell spread in 2D culture under a constant electric field (100 mV/mm) for 0 min and 20 min. Scale bar: 200 μm.

Table S2. Statistical analyses of the relationship between tumor grade, V_Tumor-Paratumor,_ and tumor spread.

| Group | Tumor grade | V_Tumor-Paratumor_ | Counts of spread/total cases | Rates of tumor spread |
| --- | --- | --- | --- | --- |
| (1) | < 2.75 (1, 2) | < -11.8 | 0 / 8 | 0 |
| (2) | < 2.75 (1, 2) | > -11.8 | 6 / 24 | 25% |
| (3) | >2.75 (3, 4) | < 50.65 | 2 / 5 | 40% |
| (4) | >2.75 (3, 4) | > 50.65 | 8 / 9 | 89% |
